# Coexpression of Sucrose Synthase and the SWEET Transporter, Which Are Associated With Sugar Hydrolysis and Transport, Respectively, Increases the Hexose Content in *Vitis vinifera* L. Grape Berries

**DOI:** 10.3389/fpls.2020.00321

**Published:** 2020-04-30

**Authors:** Ruihua Ren, Xiaofeng Yue, Junnan Li, Sha Xie, Shuihuan Guo, Zhenwen Zhang

**Affiliations:** ^1^College of Enology, Northwest A&F University, Yangling, China; ^2^Shaanxi Engineering Research Center for Viti-Viniculture, Northwest A&F University, Yangling, China

**Keywords:** *Vitis vinifera* L., photosynthates partitioning, sugar hydrolysis, sugar transport, hexose content

## Abstract

The sugar content of grape berries is affected by many factors. To explore the hexose content in different cultivars, the photosynthesis, vegetative, and reproductive biomass, as well as the enzyme activities and expression levels of genes related to sugar metabolism and sugar contents were measured. Samples were collected 70–110 days after anthesis (DAA), from Riesling (RI), Petit Manseng (PM), and Cabernet Sauvignon (CS) berries cultivated in the field. The results indicated that high expression levels of VvSWEET*15* and *VvSS3* and a high activity of sucrose synthase (SS) are associated with a higher hexose content in the berries of PM than in the berries of the other two cultivars. These genes promoted hexose accumulation in the berries by regulating sugar hydrolysis and transport. The results of this study indicate that active sugar hydrolysis and transport increase the hexose content of PM berries, which provides insights for grape berry quality improvement and breeding projects in wine production.

**Main Conclusion:** The active *VvSS3*, sucrose synthase (SS), and *VvSWEET15* increases the hexose content in Petit Manseng berries, which are associated with sugar hydrolysis and transport.

## Introduction

Plants fix inorganic C to produce carbohydrates via photosynthesis, which occurs in the mesophyll cells of mature leaves (Williams et al., 2000). After local demand is met, excess photosynthates are exported (as sucrose) from the leaves, which are in turn defined as ‘sources,’ and then arrive at non-photosynthetic organs such as young leaves, flowers, and fruit, which are defined as ‘sink-organs,’ via long-distance phloem transportation (Ludewig and Flügge, 2013). This process is necessary to maintain plant and fruit growth. Previous studies have indicated that 80% of sugar is translocated to ‘sink organs’ (Kalt-Torres and Huber, 1987). However, sugar partitioning in organs depends on development stage and ‘sink’ strength (Ho, 1988; Stitt, 1991). Vegetative growth and reproductive growth constitute the two stages of plant growth (Wardlaw, 1990). In terms of fruit-producing species, fruit is the major ‘sink’ in the reproductive growth stage, during which large amounts of photosynthates are stored in the vacuole as sucrose—such as that which occurs in tomato (Shammai et al., 2018) and apple (Zhen et al., 2018) fruit—or as hexoses (fructose and glucose), such as that which occurs in grape (Zhang et al., 2006) berries, affecting fruit quality (Kalt-Torres and Huber, 1987). Due to competition for sugar availability, sugar partitioning between vegetative growth and reproductive growth is vital with respect to increasing fruit yields and quality (Gifford et al., 1984).

Sugar transport from ‘sources’ to ‘sinks’ can be divided into three successive processes: the loading of sucrose into the ‘source’ leaf phloem; sucrose transport; and the unloading at and postphloem transport to ‘sink-organs’ (Geiger, 2011; Ludewig and Flügge, 2013). For many higher plants, it has been shown that both phloem loading and unloading are facilitated by different sugar transporters (Lucas et al., 2013), which are part of the apoplastic pathway. Inventories of sugar transporter genes have been reported in many cultivated fruit crop species (Reuscher et al., 2014; Wei et al., 2014; Li et al., 2015), most transporters are categorized as sucrose transporters (SUTs/SUCs) or monosaccharide transporters (Afoufa-Bastien et al., 2010). In the apoplastic pathway, sucrose is exported from photosynthetic mesophyll cells into the apoplast by members of the SWEET transporter family (such as *AtSWEET11* and *AtSWEET12*) (Chen et al., 2012), after which it subsequently enters companion cell and sieve element (CC-SE) complexes via SUC proteins (such as *AtSUC2*) (Gottwald et al., 2000) for loading. In addition, sugar can be transported by monosaccharide transporters (such as *VvHTs*) after sucrose is hydrolyzed into hexoses. All these processes are essential for sugar accumulation in ‘sink organs’ (Hayes et al., 2007). Sucrose phosphate synthase (SPS) contributes to sucrose accumulation at the loading site and to transportation by increasing the turgor pressure (Durand et al., 2018). Depending on their subcellular localization and substrate specificity, sucrose metabolism-related enzymes such assucrose synthase (SS) and invertase, hydrolyze sucroseinto hexose (Chen et al., 2017; Wan et al., 2018). These processes maintain sugar concentration gradients at the unloading site to maintain a high unloading rate and subsequently to translocate sugar into the vacuole (Chong et al., 2014; Lecourieux et al., 2014). Therefore, fruit is allowed to accumulate sugar rapidly (Patrick et al., 2001; Hayes et al., 2007). All of these sugar transporters, the sugar metabolism-related enzymes in the ‘sink organs,’ and sink demand, compose a complex regulatory network that determines the sugar content in ‘sinks.’

Grape is an important economic fruit crop species worldwide. Most grapes are cultivated to produce fresh fruit and for the wine-making industry, with only a fraction of grape berries being juice and dried. The sugar content of grapes is important for wine quality, both in terms of alcohol content and aroma profiles (Hayes et al., 2007). Grape growth is described as a double SS curve and consists of three stages (the first rapid-growth phase, the lag phase, and the second growth phase). However, soluble sugars (mainly hexoses) rapidly accumulate until the end of the lag phase, which indicates that the fruit is beginning to ripen or is referred to as ‘veraison’ (Coombe, 1992). The fruit sugar content varies among genotypes in plants (Liu et al., 2006; Liang et al., 2011; Zhao et al., 2016). Some genotypes with a high hexose content are used to make sweet or dry wines, which increases the alcohol content and flavor. By contrast, others may need additional sugars (Hayes et al., 2007). To date, differences in sugar contents among genotypes remain unclear, although sugar metabolism pathways are clear in plants. Therefore, three grape cultivars with distinct hexose contents were used in this study. The sugar contents and distribution between vegetative growth and reproductive growth, the enzyme activities, and the expression levels of genes involved in the sugar metabolism of berries were investigated during grape ripening. This study aims to provide a basis for understanding the differences in sugar accumulation among cultivars.

## Materials and Methods

### Materials and Design

This experiment was conducted in a commercial vineyard of *Vitis vinifera* L. cvs. ‘Riesling’ (RI), ‘Petit Manseng’ (PM), and ‘Cabernet Sauvignon’ (CS) at the ‘Yaojing’ winery located in Xiangfen County, Shanxi Province, China (35°86′56′′ N, 111°43′53′′ E, elevation 500–600 m above sea level), during the 2018 growing season. The county is located in a semi-arid, semi-humid monsoon climate zone and has a typical temperate monsoon climate. The annual average temperature is 11.5°C, the annual rainfall is 550 mm, the average temperature reaches 26°C in July, and the frost-free period lasts 185 days. During cold winters, grapevines need to be covered by soil to remain alive. The self-rooted vines in this vineyard were planted in 2013, in an east-west orientation. The spacing was 1 m × 3 m. The training system of the vines was a single cordon positioning system, pruning way was a single bud per spur. All other viticultural practices followed unified standards.

Three rows of vines were selected per cultivar and three biological replicates were established in 2018. To achieve homogeneity, each row was divided into seven plots, from which berries were collected on seven sampling dates. The plots were arranged in a completely randomized design, and each replicate was randomly sampled from 60 grape clusters of 30 plants, for a total of 300 berries. Sampling was first conducted 30 days after anthesis (DAA; the berries were pea-sized), and the second and third sampling occurred at 60 DAA (RI and CS were at veraison) and 70 DAA (PM was at veraison), after which sampling was performed at 10 days intervals until harvest (i.e., 30, 60, 70, 80, 90, 100, and 110 DAA). The berries were collected at the same time of day (9–11 a.m.), immediately frozen in liquid nitrogen, and then stored at −80°C for subsequent analysis.

### Leaf Area, Chlorophyll Content, and the Net Photosynthesis Rate

Leaf area was determined at the end of the vegetative growth phase by collecting digital images of individual leaves and analyzing them via the publicly available image analysis software ImageJ (National Institutes of Health), and the average values were obtained. The total leaf area per vine was calculated by multiplying “y” by the number of leaves per shoot, and the number of shoots per vine (Etchebarne et al., 2010; Duchêne et al., 2012). The chlorophyll concentration was determined via the method of Golan et al. (2015), with three replicates. Leaf photosynthesis was measured according to the method of Xu et al. (2018), by using an Li-Cor-6400 photosynthesis measurement system (Li-COR Inc., NE, United States) equipped with a 6-cm^2^ leaf chamber. The photosynthetically active radiation was maintained at 1,000 mmol photons m^–2^ second^–1^, and the reference CO_2_ concentration was approximately 400 mmol CO_2_ mol^–1^ air (mg mL^–1^). Six leaves per cultivar from the 6th nodes to the −9th nodes of the shoots were selected and measured between 9: 00 a.m. and 12: 00 p.m. on sunny days, at four different developmental stages. The average of three biological replicates was ultimately obtained.

### Stem Diameter, Yield, Weight at Winter Pruning

For each cultivar, 30 shoots that grew consistently were selected as materials for measuring the stem diameter at the end of the vegetative growth period. At harvest, the fruit of 30 vines were collected for each cultivar and subsequently weighted by a scale, and the yield was recorded in kilograms per vine. During winter pruning, the shoots of 30 vines were collected and immediately weighed for growth assessment. All the results were recorded as the average of three biological replicates.

### Monitoring of Berry Maturation and HPLC Analysis of Hexose Contents in the Berries

The total soluble solids (TSS) (°Brix) of berries were determined via a pocket refractometer, and the pH was determined by a pH meter to monitor fruit ripening. The berry hexose contents were obtained according to a modified method of Eyduran et al. (2015). Each sample was ground into a fine powder in liquid nitrogen. The grape powder was dissolved in water and then centrifuged at 10,000 rpm for 10 min at 4°C (5804 R centrifuge; Eppendorf AG, Hamburg, Germany), after which 1 mL of the supernatant was transferred to a new tube. Deionized water was ultimately added to bring the volume to 5 mL. All of the berry supernatants were subsequently filtered through a 0.22-μm luer syringe-filter (Biofount, Beijing, China).

The hexose contents were analyzed with an Agilent 1260 Infinity HPLC system (Milford, MA, United States) by injecting 20 μL of the supernatant into an Athena C18 column (4.6 × 250 mm, 5 μm; the column temperature was 40°C). As the mobile phase for metabolite separation, chromatographic acetonitrile (80%, v/v) was maintained at a flow rate of 1.2 mL min^–1^. Glucose and fructose relative standard curves were used for comparison to accurately calculate the sugar concentration in grams per liter. All chemicals and standards were purchased from Sigma (St. Louis, MO, United States) and were dissolved in deionized water. Each cultivar included three biological replicates.

### Enzyme Extraction and Analysis of Enzyme Activity

Enzymes involved in sugar metabolism were extracted according to the method of Keller and Ludlow (1993), with minor modifications. Each sample (consisting of three biological replicates) of 15 berries was ground to powder as described in section 2.4, and then 1 g of the sample was removed and added to 5 mL of extraction buffer three times; the mixture was further ground into a homogeneous liquid for 3–5 min on ice. The buffer used consisted of 100 mmol L^–1^ Tris-HCl (pH 7.0), which consisted of 5 mmol L^–1^ MgCl_2_, 2 mmol L^–1^ ethylenediaminetetraacetic acid disodium salt (EDTA-Na_2_), 2% ethylene glycol, 0.2% (w/v) bovine serum albumin (BSA), 2% polyvinyl pyrrolidone, and 5 mmol L^–1^ dithiothreitol. The homogenates were then centrifuged (at 10,000 rpm at 2°C for 20 min). Each supernatant was immediately transferred to a new tube and stored at −20°C until analysis. All procedures were performed at 0–4°C.

#### Activity of SS and SPS

The enzyme reaction solution to determine SS activity consisted of 100 mmol L^–1^ Tris-MES buffer (pH 7.0), which comprised 10 mmol L^–1^ D-fructose, 5 mmol L^–1^ magnesium acetate, and 5 mmol L^–1^ dithiothreitol. Each mixture, which included 0.4 mL of enzyme reaction solution, 0.1 mL of 10 mmol L^–1^ uridine diphosphate glucose (UDPG) and 50 μL of the supernatant of the enzyme extraction solution, was added to 1 mL of deionized water. Each mixture was then incubated at 30°C for 10 min and subsequently placed in boiling water for 3 min to terminate the reactions. Afterward, 0.1 mL of 2 mmol L^–1^ NaOH was added, and the mixtures were incubated in boiling water for 10 min. After cooling, 3.5 mL of 30% HCl and 1 mL of 0.1% resorcinol were added, and the mixtures were subsequently incubated in an 80°C water bath for 10 min. Afterward, the color development was measured at 480 nm to determine the sucrose content. A control tube containing 50 μL of water instead of UDPG was also measured, and the difference was used to calculate the SS activity. The procedure to analyze the SPS activity was the same as that for SS except that 10 mmol L^–1^ fructose-6-phosphate (F6P) was used in enzyme reaction mixtures.

#### Analysis of AI and NI Activity

The enzyme reaction solution to determine AI activity consisted of 80 mmol L^–1^ potassium phosphate-HAc buffer (pH 4.7) and 50 mmol L^–1^ sucrose. The mixtures, which included 0.95 mL of enzyme reaction solution and 50 μL of the supernatant of the enzyme extraction solution, were transferred into a new tube and incubated for 10 min at 30°C, after which they were stopped by being placed in boiling water for 3 min. After cooling, 1 mL of 3,5-dinitrosalicylic acid was added, and the mixtures were incubated in boiling water for 5 min. After the mixtures cooled to room temperature, their color development was measured at 540 nm. Mixtures containing 50 μL of the supernatant of inactivated enzyme extraction solution were used as the control group, and the other steps were similar to those described above; the difference was used to calculate the AI activity. Reaction mixtures for the NI activity contained 0.95 mL of 80 mmol L^–1^ potassium phosphate-HAc buffer (pH 7.0), 50 mmol L^–1^ sucrose, and 50 μL of the supernatant of the enzyme extraction solution, and the rest of the procedure was similar to that for AI. The enzyme activity was expressed as milligrams of sucrose per gram of fresh weight (FW) per hour.

### Preparation of Total RNA and cDNA and RT-PCR Analysis

Total RNA was isolated from ground berries by removing the seeds using a General Plant Total RNA Extraction Kit (Bioteke, Beijing, China) and purified with RNase-free DNaseI to remove any contaminating gDNA. The integrity of the RNA was determined using a Bioanalyzer 2100 (Agilent). The total RNA concentration was measured using a spectrophotometer (BioDrop, Cambridge, England, United Kingdom) and further verified by 1% formaldehyde-agarose gel electrophoresis. First-strand cDNA was synthesized using HiScript II Q RT SuperMix (Vazyme, Nanjing, China) according to the manufacturer’s instructions. The real-time quantitative PCR (qPCR) primers used were synthesized by Biotech Co., Ltd. (Shanghai, China) and checked in advance via melting curve analysis and electrophoresis to avoid dimer formation and non-specific amplification. Details of the primers used are listed in Supplementary Table 1.

The qPCR reaction system (10 μL) was prepared using 2× ChamQ^TM^ SYBR qPCR Master Mix (Vazyme: Q311-02), which consisted of 5 μL of 2× SYBR qPCR Master Mix, 0.4 μL of each primer (10 μM), 1 μL of 2 μg μL^–1^ diluted cDNA, and 0.2 μL of 50× ROX Reference, and RNase-free water was added to the total volume. The expression of three replicates was subsequently analyzed using the QuantStudio^TM^ 5 Real-Time PCR Instrument (96-well; 0.2 mL well^–1^). The steps of the reaction were as follows: an initial denaturation at 95°C for 30 s, followed by 40 cycles of amplification (denaturation at 94°C for 10 s, followed by extension at 60°C for 30 s). The relative expression levels of the genes were normalized to those of *VvActin* and calculated according to the 2^–ΔCT^ method, where ΔCT = CT target-CT *VvActin* (Xie et al., 2015). Three biological replicates were analyzed for each cultivar.

### Statistical Analysis

All the data are expressed as the means ± standard deviations (SDs) of triplicate experiments, and analysis of variance (ANOVA) was conducted to determine the significant differences at *P* < 0.05. Excel 2013 and SPSS 20.0 (IBM, NY, United States) were used for calculations and statistical analyses. Figures were constructed via Origin Pro 2016 (OriginLab, MA, United States) and GraphPad Prism 7.0 (Emerald Biotech Co., Ltd, Hangzhou, China). Hierarchical cluster analysis was conducted via TB-tools software.

## Analysis of the Results

### Leaf Area, Chlorophyll Content, and Photosynthesis Assessment of Different Cultivars

**FIGURE 1:**
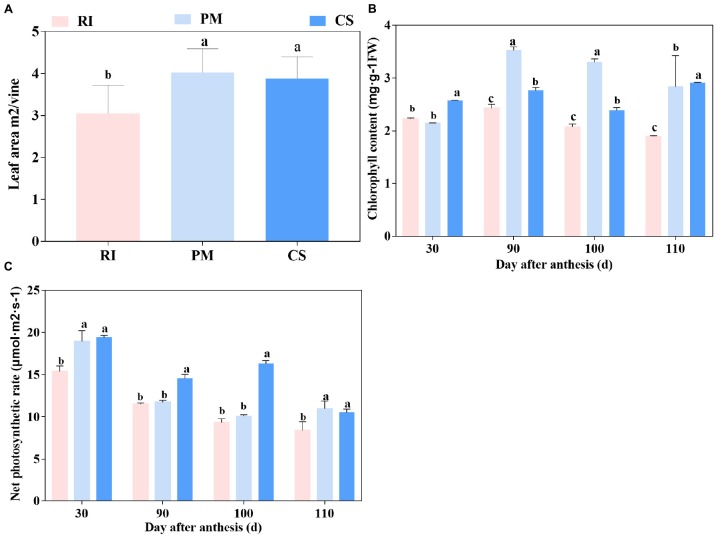
The photosynthetic capacity of different genotypes. The vine’s photosynthetic capacity is shown by leaf area **(A)**, chlorophyll content **(B)** and net photosynthetic rate **(C)** in three biological replicates. The pink columns represent Riesling (RI), the light blue columns represent Petit Manseng (PM), the dark blue columns represent Cabernet Sauvignon (CS). Column value is shown by mean ± SD. Error bars represent the SD of the means. Different letters (a, b, c) show significantly different at *P* < 0.05 level.

In this study, the photosynthesis parameters of three cultivars were measured. The leaf area per vine ranked in the order of PM > CS > RI (Figure 1A). The chlorophyll content tended to increase from 30 to 90 DAA but then decreased until 110 DAA for the three cultivars, with the exception of CS at 110 DAA (Figure 1B). The chlorophyll content was significantly higher in PM than in RI and CS at both 90 and 100 DAA. The leaf net photosynthesis rate decreased gradually during grape ripening (Figure 1C). Among the leaf net photosynthesis rate of the cultivars, the leaf net photosynthesis rate of PM and CS were significantly higher than those of RI at 30 and 110 DAA, and the leaf net photosynthesis rate of CS was the highest at 90 and 100 DAA (Figure 1C).

### Photoassimilate Partitioning Between the Vegetative Growth and Reproductive Growth of Different Cultivars

**FIGURE 2:**
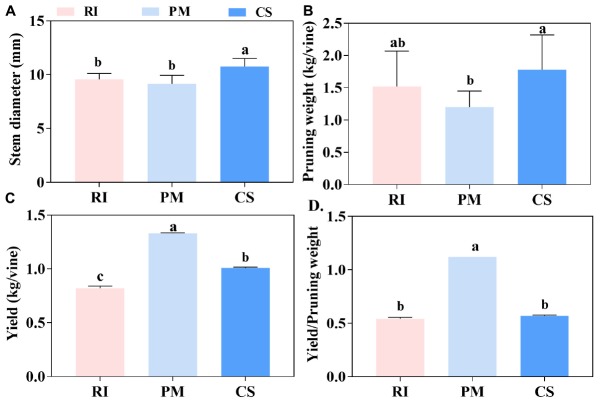
The vegetative growth and reproductive growth of different genotypes. The vegetative growth of vines is shown by stem diameter **(A)** and pruning weight **(B)**, the reproductive growth of vines is shown by yield **(C)**, and the yield/pruning weight shows the photoassimilates partitioning between the vegetative growth and reproductive growth **(D)** in three biological replicates. The pink columns represent Riesling (RI), the light blue columns represent Petit Manseng (PM), the dark blue columns represent Cabernet Sauvignon (CS). Column value is shown by mean ± SD. Error bars represent the SD of the means. Different letters (a, b, c) show significantly different at *P* < 0.05 level.

Distinct growth characteristics were observed among the different cultivars. The stem diameter of CS was significantly higher than that of RI and PM (Figure 2A). Similarly, pruning weight was the highest for CS, followed by RI and PM (Figure 2B). In contrast, the highest yield was observed for PM, whereas the lowest was observed for RI (Figure 2C). Last, the ratio of yield to pruning weight was highest for PM (1.12) and lowest for RI (0.54) (Figure 2D).

### Monitoring of Fruit Ripening

**FIGURE 3:**
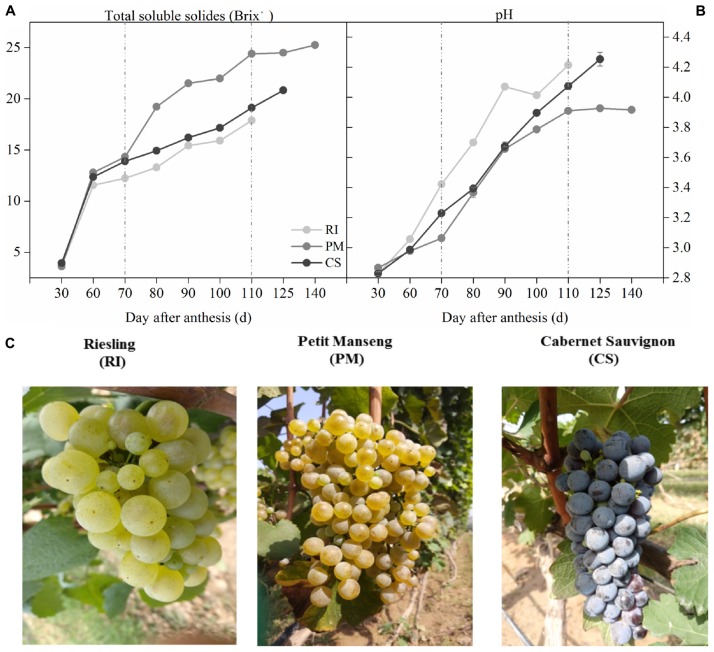
The process of grape berries ripening among different genotypes. Images show the samples ripening collected at 30, 60, 70, 80, 90, 100, 125, and 140 DAA (day after anthesis), by the soluble solids **(A)** and pH **(B)** in three biological replicates. Image **(C)** shows the grapes at harvest. The light gray dots represent Riesling (RI), the dark gray dots represent Petit Manseng (PM), the black dots represent Cabernet Sauvignon (CS). Column value is shown by mean ± SD. Error bars represent the SD of the means.

The three cultivars in this study had different maturation times, and that of PM was the latest. The TSS increased with the development of the berries and reached a maximum at harvest (Figure 3A), which was similar to that which occurred for the pH values (Figure 3B). Figure 3C shows the three grape cultivars at harvest. The growth curve shows a significant difference in TSS between the cultivars beginning at 70 DAA and a minor change in PM after 110 DAA. Therefore, samples from 70 to 110 DAA were selected for further analysis.

### Hexose Content of the Berries During Ripening

**FIGURE 4:**
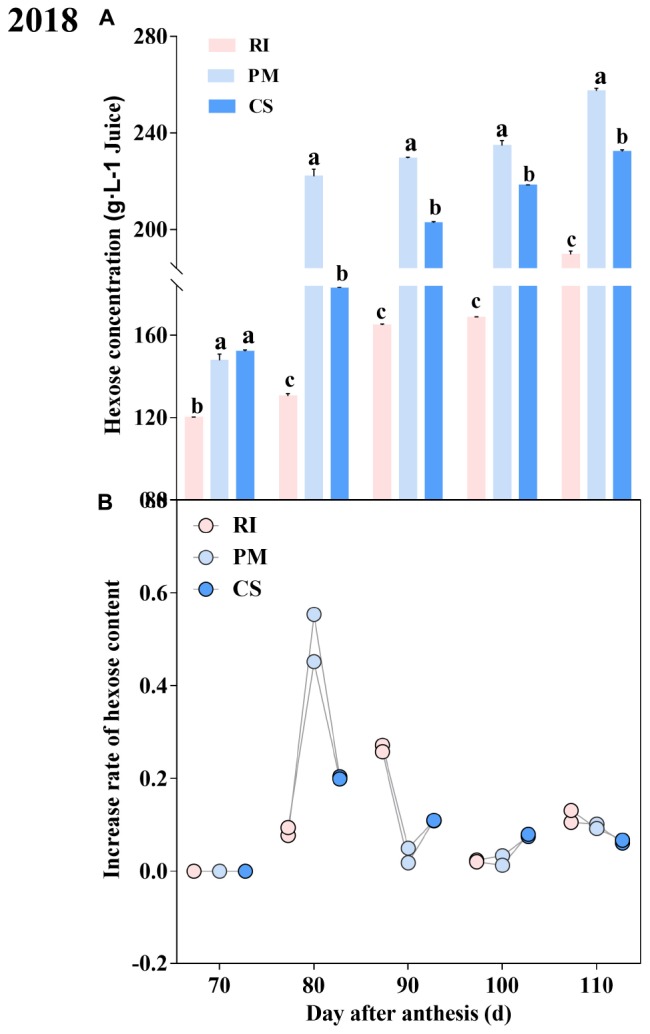
The dynamic hexose accumulation and accumulation rate during ripening Hexose content in the berry **(A)** and the accumulation rate in berry **(B)** at 70, 80, 90, 100, and 110 DAA, in three biological replicates. The pink columns and dots represent Riesling (RI), the light blue columns and dots represent Petit Manseng (PM), the dark blue columns and dots represent Cabernet Sauvignon (CS). Column value is shown by mean ± SD. Error bars represent the SD of the means. Different letters (a, b, c) show significantly different at *P* < 0.05 level.

The hexose content in the berries at different growth stages was investigated. The hexose content exhibited distinct dynamic patterns during ripening. The hexose content in the berries increased gradually with development and reached a maximum at 110 DAA (Figure 4A), owing to increases in both fructose and glucose (Supplementary Table 2). The hexose contents during ripening were PM > CS > RI. The sugar rapidly accumulated in the PM and CS berries at 80 DAA but accumulated at 90 DAA in the RI berries (Figure 4B). The hexose accumulation rate of the PM berries was evidently 7.19- and 2.73-fold that of the RI and CS berries at 80 DAA, respectively.

### Analysis of Enzyme Activity in the Berries During Ripening

**FIGURE 5:**
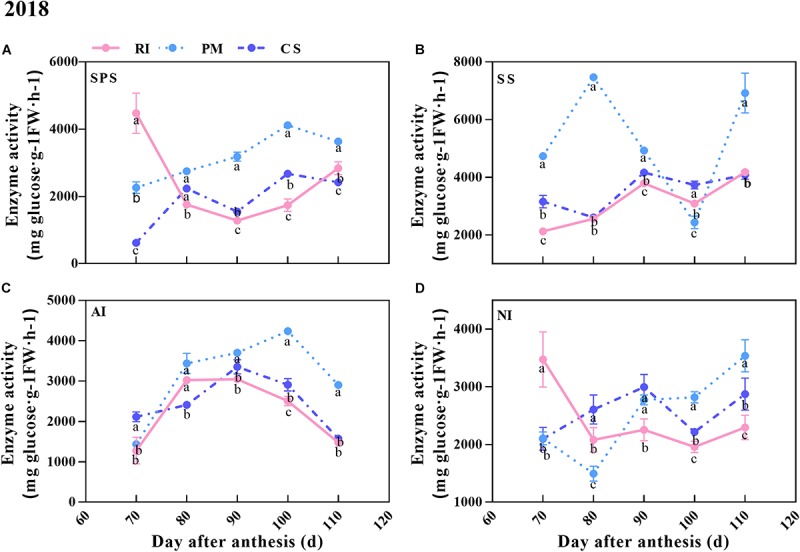
The activities of sugar metabolism-related enzymes during ripening. The SPS, SS, AI, and NI activities in the berry at 70, 80, 90, 100, and 110 DAA, in three biological replicates. The pink line represent Riesling (RI), the light blue line represents Petit Manseng (PM), the dark blue line represents Cabernet Sauvignon (CS). The dot value is shown by mean ± SD. Error bars represent the SD of the means. Different letters (a, b, c) show significantly different at *P* < 0.05 level.

In this study, the activities of sucrose metabolism-related enzymes (SPS, SS, AI, NI) in the berries of the three cultivars were measured. The results showed that the activity patterns of SPS and NI were similar, as they increased overall in the berries of the three cultivars during ripening (Figures 5A,D). The AI activity increased in the early stage but decreased from 100 to 110 DAA (in the PM berries) or from 90 to 110 DAA (in the RI and CS berries) (Figure 5C). As shown in Figure 5, although the activities of SPS and AI were higher in the PM berries than in the CS and RI berries at 80–110 DAA, they were still less than the activity of SS in the PM berries at all time points except 100 DAA. The SS activity in the PM berries increased to the maximum at 80 DAA and was 2.83- and 2.81-fold that in the RI and CS berries, respectively (Figure 5B). However, the SS activity decreased from 80 to 100 DAA in the PM berries and was close to the value of both the RI and CS berries. The high SS activity was consistent with the high hexose accumulation rate at 80 DAA (Figures 4B, 5B). The NI activity varied dynamically in the berries of the three cultivars and was high in the PM berries at 90–110 DAA (Figure 5D).

### Expression Patterns of Genes Related to Sugar Metabolism and Transport in Berries During Ripening

**FIGURE 6:**
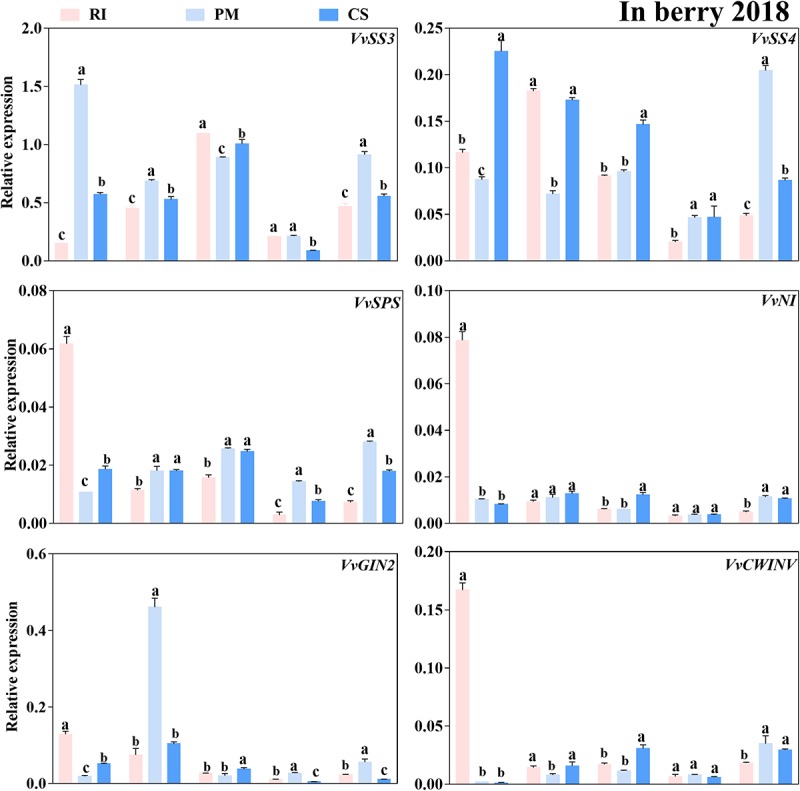
The relative expression levels of sugar metabolism-related enzyme genes in the berry. The gene expression levels in the Riesling (RI, pink columns), Petit Manseng (PM, light blue columns), and Cabernet Sauvignon (CS, dark blue columns) berries at 70, 80, 90, 100, and 110 DAA, in three biological replicates. Column value is shown by mean ± SD. Error bars represent the SD of the means. Different letters (a, b, c) show significantly different at *P* < 0.05 level.

**FIGURE 7:**
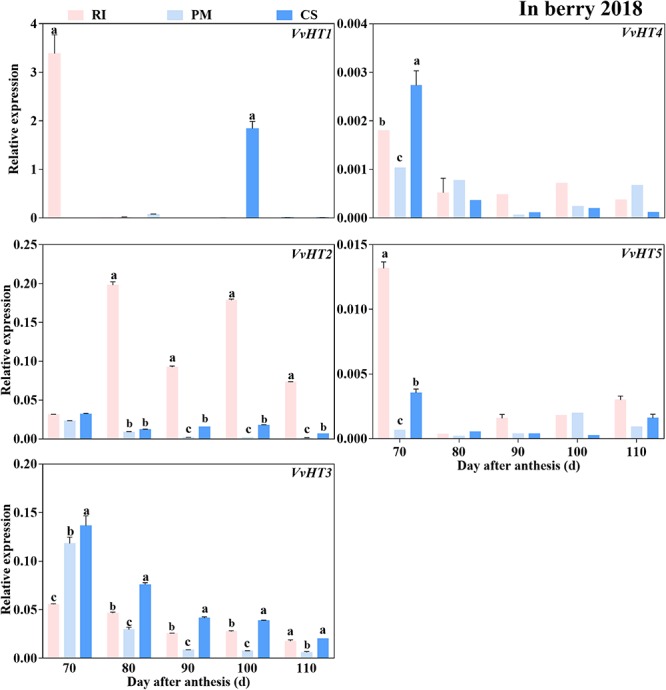
The relative expression levels of hexose transporter genes (*VvHTs*) in the berry. The gene expression levels in the Riesling (RI, pink columns), Petit Manseng (PM, light blue columns), and Cabernet Sauvignon (CS, dark blue columns) berries at 70, 80, 90, 100, and 110 DAA, in three biological replicates. Column value is shown by mean ± SD. Error bars represent the SD of the means. Different letters (a, b, c) show significantly different at *P* < 0.05 level. The lack of statistical analysis is due to the low expression value of the gene analyzed (in some time points).

**FIGURE 8:**
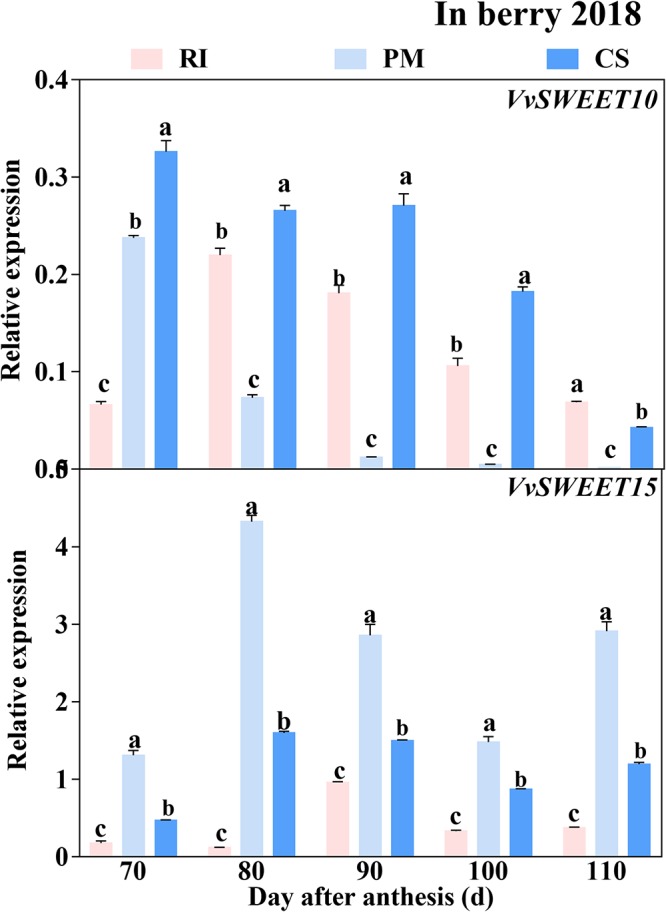
The relative expression levels of *VvSWEETs* in the berry. The gene expression levels in the Riesling (RI, pink columns), Petit Manseng (PM, light blue columns), and Cabernet Sauvignon (CS, dark blue columns) berries at 70, 80, 90, 100, and 110 DAA, in three biological replicates. Column value is shown by mean ± SD. Error bars represent the SD of the means. Different letters (a, b, c) show significantly different at *P* < 0.05 level.

To enter their target site, sugars must be taken up from the apoplast, which is likely mediated by sucrose or hexose transporters together with activity of sugar metabolism-related enzymes. It remains unclear whether mostly sucrose or hexose produced from sucrose hydrolysis via sugar metabolism-related enzymes is imported into sink organs by sucrose or hexose transporters. It is also a mystery which transporter is mainly responsible for this process. Therefore, the expression levels of the genes encoding these proteins in the berries of three cultivars were analyzed at different developmental stages in this study (Figures 6–8). Six genes coding sucrose metabolism-related enzymes exhibited different expression patterns in the berries among the cultivars. The expression level of *VvSPS* was low and below 0.03 at all stages (except 70 DAA), despite being highly expressed in the PM berries at 100 and 110 DAA (Figure 6). Similarly, the *VvNI* and *VvCWINV* expression levels were also low (below 0.05), except at 70 DAA, so the biological significance associated with the difference in gene expression among cultivars diminished. However, *VvSS3* was the most highly expressed gene in the berries. Compared to that in the RI and CS berries, the *VvSS3* expression level in the PM berries was higher at 70, 80, and 110 DAA (Figure 6), and the *VvGIN2* and *VvSS4* expression levels were higher at 80 and 110 DAA, respectively (Figure 6). Moreover, the *VvGIN2* expression levels in the PM berries were 6.17- and 4.43-fold those in the RI and CS berries at 80 DAA, respectively, and the *VvSS4* expression levels were 4.2- and 2.37-fold those in the RI and CS berries at 110 DAA, respectively. The *VvSS3* expression level and SS activity were higher in the berries than that of other enzymes, although those in berries reduced during late stages of ripening.

Similar to the expression trend of the genes encoding sugar metabolism-related enzymes, the genes encoding sugar transporters were highly expressed in the early ripening stage. The *VvHT2* and *VvHT3* members of the *VvHT* family were highly expressed in the berries. However, the expression levels of *VvHT4* and *VvHT5* (except at 70 DAA) were extremely low and below the threshold of 0.01 minimal relative expression with biological significance during ripening (Figure 7), and the expression of *VvHT1* was detected only in RI berries at 70 and in CS berries at 90 DAA. Among the cultivars, the expression level of *VvHT2* was highest in the RI berries from 80 to 110 DAA, and that of *VvHT3* was highest in the CS berries at 70–100 DAA (Figure 7). Seven members of the SWEET family that were highly expressed in grape berries (Chong et al., 2014) were investigated in this study (Supplementary Table 3), and the results showed that *VvSWEET10* and *VvSWEET15* were differentially expressed among the cultivars. Importantly, *VvSWEET15* was highly expressed in the PM berries during ripening (Figure 8). At 80 DAA, the *VvSWEET15* expression level in the PM berries was 36- and 2.7-fold that in the RI and CS berries, respectively, in which a high hexose accumulation rate was maintained (Figures 4B, 8). By contrast, *VvSWEET10* was highly expressed in the CS and RI berries from 80 to 110 DAA (Figure 8).

### Correlation Analysis

**FIGURE 9:**
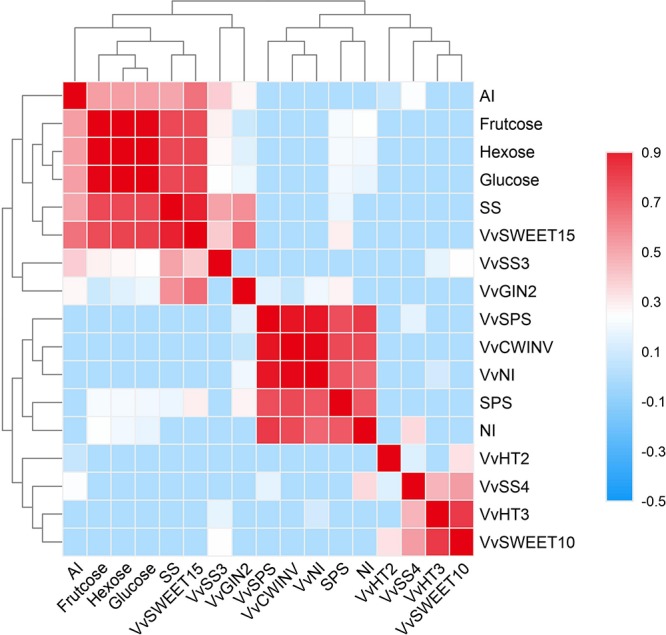
The correlation analysis of the sugar contents associated with sugar metabolism-related enzymes and genes, and the sugar transporter genes. The correlation in the Riesling (RI), Petit Manseng (PM), and Cabernet Sauvignon (CS) berry during ripening.

Sugar content is closely related to sugar metabolism and transport in plants. Final sugar content is usually determined by many enzymes and genes that interact with and regulate each other. Heatmaps were constructed that show that sugar contents were correlated with both sugar metabolism-related enzymes/genes and with the genes encoding sugar transporters in the berries during grape ripening (Figure 9). The correlation coefficients were expressed by colored segments at the corresponding locations, and these indicators were assigned to three different groups (Figure 9). As shown in Figure 9, all sugars as well as the sucrose metabolism-related enzymes SS, *VvSS3*, and *VvSWEET15* were categorized into the same group in the berries, which suggested that the sugar content in the berries was closely related to changes in SS activity, *VvSWEET15* and *VvSS3* expression.

## Discussion

### The Photosynthesis Rate of the Cultivar With High Hexose Content Is Moderate

Photosynthates, which were hydrolyse to hexoses (fructose and glucose), from leaf photosynthesis are the source of sugar accumulation in grape berries (Coombe and Mccarthy, 2000). Fruit trees with strong photosynthesis can fix more assimilated carbon and produce high-sugar-containing fruit (Ainsworth and Bush, 2011). The results of this study showed that CS, with a moderate hexose content, had the strongest photosynthesis capability among the three cultivars (Figures 1C, 4A). Although it had large photosynthetic area and a high chlorophyll content (Figures 1A,B), PM, with a high hexose content, presented a moderate level of photosynthesis from 90 to 100 DAA (Figures 1C, 4). Previous studies reported that RuBisCO activase and nitrogen content have an impact on the photosynthesis capability of different species (Benomar et al., 2019). Additionally, excess sugar that accumulates in the leaves also suppresses RuBisCO activity (Hdider and Desjardins, 1995) and influences photosynthesis. This means that a combination of factors coordinately regulates plant photosynthesis.

### Greater Amounts of Photosynthetic Products for Fruit Growth Are Available in Cultivars With a High Hexose Content

Mesophyll cells in the leaves fix CO_2_ to produce photosynthates, which is the first step for plant survival. However, photosynthate partitioning between leaves and heterotrophic plant organs strongly determines plant growth and development, as well as crop yield (Génard et al., 2008). Photosynthates are transported to sink organs that demand energy (such as fruit) via long-distance transport in the phloem (Pastenes et al., 2014). Previous studies have indicated that there is a regulatory mechanism that regulates the balance between vegetative growth and reproductive growth as well as the relationship between sink organs and source organs (Pastenes et al., 2014). The results of this study showed that CS and RI presented a greater stem diameter and pruning weight than did PM (Figures 2A,B). It’s likely that more leaf photosynthates flowed to the plant for vegetative growth during the growing season in CS and RI. By contrast, PM obtained high yields at harvest (Figure 2C). Therefore, it was obvious that the highest ratio of yield to pruning weight occurred in PM (Figure 2D). This result suggested that the CS and RI cultivars preferentially translocated photosynthates for vegetative growth, which was in accordance with the strong shoot growth, while PM preferentially underwent reproductive growth to increase yields. It remains unclear whether this means that the phloem transport of sugar from the leaves to the fruit is more active in PM than in CS and RI.

### Invertase and Hexose Transporters Are Unnecessary for Sugar Differential Accumulation Among Cultivars

Grape berries accumulate hexoses, which means that sucrose imported into sink organs must be cleaved via sucrose metabolism. Sucrose metabolism has been studied extensively in several species and is necessary for phloem unloading to continue, as well as for the accumulation of additionally assimilated carbon in the ‘sinks.’ Therefore, sucrose-cleaving enzymes play a vital role in sink growth. Invertases, including acidic (CWINV or GINS) and neutral (NI) invertases, are reported to be responsible for such cleavage. CWINV, located in the cell wall, is defined as a sink-specific enzyme (Coninck et al., 2005) and functions in phloem unloading in plants (Wan et al., 2018). In tomato, *SlCWINV1* is highly expressed in the fruit, and its product is involved in the apoplastic unloading of sucrose (Jin et al., 2009). However, Haves et al. (2007) reported that *VvCWINV* was expressed at a low level in grape berries after veraison, and a similar phenomenon for *MdCWINVs* was reported in apple (Haves et al., 2007; Li et al., 2012). It has been suggested that CWINV may not be necessary for apoplastic sucrose unloading in fruit (Nardozza et al., 2013). These results were further confirmed in this study. Here, the *VvCWINV* transcript levels were below 0.1 during ripening (except in RI berries at 70 DAA), and the enzyme activity was relatively low. Similarly, GINS and NI also were expressed at low levels and presented low enzyme activities. These results suggested that sugar metabolism may be conserved within species (Dai et al., 2016), that invertase (CWINV) is expressed mainly in berries before veraison for cell division and growth, that gene expression and enzyme synthesis (GINs) may occur earlier than the accumulation of hexoses in berries, and that (NI) maintains cytosolic sugar homeostasis for cellular function (Barratt et al., 2009; Wan et al., 2018). Therefore, other mechanisms must be involved in sugar accumulation in fruit (Davies and Robinson, 1996).

Hexose transport is an important part of sugar transport to sink organs due to the existence of sucrose metabolism. Hexose transporters (HTs) have been studied in many crop species (Afoufa-Bastien et al., 2010; Wang et al., 2019) and are related to fruit sugar content (Wang et al., 2019). In contrast, fruit hexose import is inhibited and yield decreased in antisense mutants of *LeHT2* and *LeHT3* (McCurdy et al., 2010). Six full-length cDNAs (*VvHT1*-*VvHT6*) have been cloned from grape (Afoufa-Bastien et al., 2010), and *VvHT6*, whose sequence is highly homologous with that of TMTs, was identified as *VvTMT1*. As described in the reference, here, the expression levels of *VvHT5* and *VvHT4* were extremely low (below 0.01 at most stages), and *VvHT1* expression was not detected at most stages in the three cultivars (Afoufa-Bastien et al., 2010). These results could be explained by *VvHT1* being highly expressed in young berries to provide energy for growth and *VvHT5* being expressed in seeds and mature leaves (Vignault et al., 2005; Hayes et al., 2007). In contrast, the high relative expression levels of *VvHT2* in RI berries and *VvHT3* in CS berries after veraison seem to be consistent with the shift in phloem unloading in those berries after veraison (Lecourieux et al., 2014). However, there were no significant correlations between the expression levels of *VvHTs* and hexose content during grape ripening (Figure 9), as shown by Hayes et al. (2007). These results suggested that multiple pathways are employed for sugar accumulation and that HTs are responsible for a small part of fruit sugar transport (Ludewig and Flügge, 2013). In addition, the transport characteristics of HT2 and HT3 are unknown in plants (Hayes et al., 2007), and a functional characterization experiment would help explore the roles of *VvHT2* and *VvHT3* in assimilating importation into sink organs.

### Coexpression of SS and SWEET Genes Enhances the Hexose Content in PM Berries

Phloem unloading is indispensable to sugar transport for supporting sink organs (such as developing seeds and fruit) and has been confirmed to undergo a switch from the symplastic route to the apoplastic route in berries at veraison (Zhang et al., 2006), as reported in Arabidopsis ovules (Werner et al., 2011). The apoplastic pathway requires an active transport process, where sugar transporters transport sugar into the apoplastic space and subsequently into parenchyma cell sinks (Davies et al., 1999), independent of turgor pressure differences. This ensures the efficiency of phloem transport and is critical for sugar accumulation in fruit (Zhang et al., 2006; Ludewig and Flügge, 2013). Members of the SWEET family have been identified as sugar efflux transporters (Chen et al., 2012). Extensive studies have indicated that abundant SWEETs are vital to the physiological development of plants, inclusive of processes such as phloem loading (Chen et al., 2012), phloem unloading (Ho et al., 2019), the response to cold stress (He et al., 2008), and seed filling (Ma et al., 2017). Chen et al. (2015) reported that SWEET15, a plasma membrane or intracellular protein, functions as a sucrose exporter in *Arabidopsis*. *AtSWEET15* is involved in sugar transport from the phloem to sinks in *Arabidopsis*, such as the release of sucrose from the seed coat into the apoplasm to supply seed filling (Chen et al., 2015) and the response to osmotic stress in the roots (Durand et al., 2016, 2018). In the present study, *VvSWEET15*, a paralog of *AtSWEET15* (Chong et al., 2014), was highly expressed in the berries of the three cultivars during ripening, as described in Chardonnay berries (Zhang et al., 2019). The expression levels of *VvSWEET15* were highest in the PM berries, with a high hexose content, and were significantly positively correlated with berry hexose content during ripening. These results could be explained by the locations on chromosomal regions harboring QTLs for sugar content and those related to the sugar content (Zhen et al., 2018). The strongly expressed *VvSWEET15* in PM berries may be involved in releasing sucrose into the apoplastic space (Asai et al., 2016) and promoted sugar accumulation in berries. These results also supported that the knockout of *GmSWEET15* significantly reduced the sucrose and glucose content in the embryos and increased seed abortion rates (Wang et al., 2019). The upregulation of *VvSWEET15* expression levels detected in this study cannot exclude the following factors: (1) Sugar is imported into grape berries after veraison, and this increased sink needs to drive the expression of a variety of sugar transporters to activate sugar phloem transport to the fruit (Lecourieux et al., 2010, 2014); (2) It has been suggested that sugars can act as molecular signals regulating fruit development and gene expression in plants. Sugar transporters are induced by both glucose and sucrose and activate sugar response elements within promoter regions (Atanassova et al., 2003). Therefore, gene expression is regulated by substrates cannot be excluded in this study.

SS is involved in sucrose hydrolysis in the cytosol and is necessary for fruit growth (Braun et al., 2014). Here, both the gene expression level and the enzyme activity of SS were higher than those of invertase in the berries at most stages after veraison, which was consistent with the results of kiwifruit (Moscatello et al., 2011) and citrus (Katz et al., 2011). This further supported the idea that SS is much more important than invertase for sugar accumulation (Dreier et al., 1998; Chen et al., 2017) and that SS increases hexose contents in litchi cultivars (Yang et al., 2013), potato (Hajirezaei et al., 2000), and grape (Wu et al., 2011). In this study, the *VvSS3* expression levels and SS activities in the PM berries, with the highest hexose content, were higher than those in the RI and CS berries at 70, 80, and 110 DAA. In the cytosol, sucrose is hydrolyzed into hexose to decrease sucrose accumulation and thus promote sugar transport (Zhu et al., 2017), as was reported by Zhang et al. (2013) for *PpSUS1* in peach. However, the low expression of *VvSS4* in the berries may be caused by tissue-specific expression in flowers (Zhu et al., 2017). In conclusion, *VvSWEET15* and *VvSS3* were highly expressed in berries after veraison when hexoses began to accumulate, which seems to be consistent with the idea that sucrose metabolism-related enzymes cleaves sucrose that is unloaded into the apoplastic space into hexoses, which reduces sucrose concentrations at the unloading site and promotes sucrose transport (Hayes et al., 2007; Moscatello et al., 2011).

Gene transcription and subsequent translation jointly determine the activity of proteins. Previous studies have indicated that gene expression is regulated by developmental (Afoufa-Bastien et al., 2010) and many environmental factors (Medici et al., 2014). Here, the expression level of *VvSS3* and SS activity decreased during late ripening (especially at 100 DAA), which may be due to rainfall at that stage (Duan et al., 2019). Moreover, gene expression levels decreased during late ripening (Li et al., 2012), ultimately reducing the hexose accumulation rate (Figure 4A). However, these did not affect the highest hexose content in the PM berries at harvest because hexoses accumulated largely in those berries at 80 DAA (Figure 4B). In addition, enzyme activity depends on both translational and posttranslational activation, which means that gene expression is never entirely consistent with enzyme activity (Williams et al., 2000; Wormit et al., 2006). It is likely that protein translation and activation occurred later than gene expression did (Duan et al., 2019), as *VvSS3* was strongly expressed in PM berries at 70 DAA, but the activity of SS and the hexose accumulation rate were highest at 80 DAA (Figures 4–6).

## Conclusion

After veraison, *VvSWEET15* and *VvSS3* highly expressed in the grape berries when hexose began to accumulate. The strong expression of the *VvSWEET15* gene and high SS activity increased the hexose accumulation rate in the PM berries at 80 DAA. These phenomena eventually caused the highest hexose content to occur in the PM berries rather than in the RI and CS berries. At harvest, relatively strong reproductive growth occurred for the PM vines. Together, these results indicate that the active *VvSS3* and *VvSWEET15* genes in the PM berries could promote fruit sugar accumulation by affecting sugar hydrolysis and transport. This study provides knowledge for understanding the intra-species diversity of fruit sugar content, although the regulatory mechanism of genes responding to sugar accumulation remains unclear. It must be emphasized that the gene expression levels and enzyme activities studied in this paper represent only the average levels for whole berries, so advanced methods could contribute to the precise tracking of events of sugar metabolism and transport in specific phloem locations in the future. Moreover, the functional characterization of related genes and identification of transcription factors related to phloem transport are needed to unravel the mystery of sugar import in fruit.

## Data Availability Statement

The datasets generated for this study are available on request to the corresponding author.

## Author Contributions

RR and ZZ conceived the original screening and research plans. ZZ supervised the experiments. RR performed the experiments in the lab, carried on the field experiments, designed the experiments, analyzed the data, and edited the manuscript. SX, XY, and SG provided technical assistance to RR. JL provided the technical assistance to RR. XY provided language assistance to RR. RR and ZZ conceived the project and wrote the manuscript with contributions of all the authors.

## Conflict of Interest

The authors declare that the research was conducted in the absence of any commercial or financial relationships that could be construed as a potential conflict of interest.

## Abbreviations

AI: acid invertase
CC-SE: companion cell and sieve element
CS: Cabernet Sauvignon
CWINV: cell wall invertase
DAA: day after anthesis
GIN: vacuolar invertase
MSTs: monosaccharide transporters
NI: neutral invertase
PM: Petit Manseng
RI: Riesling
SPS: sucrose phosphate synthase
SS: sucrose synthase
SUTs/SUCs: sucrose transporters.
