# Supplementary material for: Coexpression of Sucrose Synthase and the SWEET Transporter, Which Are Associated With Sugar Hydrolysis and Transport, Respectively, Increases the Hexose Content in Vitis vinifera L. Grape Berries
Source: Front Plant Sci. 2020 Apr 30;11:321. doi: 10.3389/fpls.2020.00321 (PMC7221319; doi:10.3389/fpls.2020.00321)
Supplement: Supplementary file 1 [file Table_1.docx]

**Coexpression of sucrose synthase and the SWEET transporter, which are associated with sugar hydrolysis and transport, respectively, increases the hexose content in *Vitis vinifera* L. grape berries**

**Fronters in plant science**

Ruihua Ren^a^, Xiaofeng Yue^a^, Junnan Li^a^, Sha Xie^a^,Shuihuan Guo^a^, Zhenwen Zhang^a,b,*^

*Corresponding author: Zhenwen Zhang, College of Enology, Northwest A&F University, No. 22 Xinong Road, Yangling 712100, Shaanxi, China, Tel: 0086-13991879905; Email: [zhangzhw60@nwsuaf.edu.cn](mailto:zhangzhw60@nwsuaf.edu.cn).

**Supplemental Table 1** List of primers for RT-PCR in this study.

| Names | Accession number | Forward primers/Reverse primers 5’-3’ |
| --- | --- | --- |
| *VvNI*  *VvCWINV*  *VvGIN2*  *VvSS3*  *VvSS4*  *VvSPS* | EU016365.1  NC_012015.3  AAB47172.1  VIT_207s0005g00750  VIT_211s0016g00470  NC_012011.3 | F : GGTCTTTATTCGGGATTTTGTC  R : TGTAGCAGTCCACAGTCTTCTC  F : GCTGGACTAAAGCACAACACC  R : CTGGGACATAGGTATCCTTCTCAC  F: GATGAAGCCACTCTATGAGGTCC  R : TAGAAGGGGTTCTTCATGCTGTC  F : GCCCTGCATGGTTCAATTGA  R : GTCAAGCCTTGCCATGGAAA  F : TGGCTACTTTGCTCAGGACA  R : GTCCTTGCTGCTTGATACGG  F : CAGGGTCGACCTCTTCACTC  R :ATATGGCCAAACAGGCTGAC |
| *VvHT1*  *VvHT2*  *VvHT3*  *VvHT4*  *VvHT5*  *VvTMT1*  *VvTMT2*  *VvSWEET4* | AJ001061  AY663846  AY538259  AY538260  AY538261  GSVIVT00036283001  GSVIVT01032489001 | F : CGTTGTTCACATCGTCGCTTTATC  R : GAGCAGTCCTCCGAATAGCATTG  F : GGCATAGGGGTGGTGGTAG  R : TCGGGCTTTACGGAGAGAG  F : GCTACTCTCTACTCGTCCGCTTTG  R : CCCGTCATTGCTTCCGAACTTG  F : GGGCTGGCGAGTTTCTCTAG  R : AGTTCTGCTTGGACATCGTTTG  F : CAGGCTGTTCCACTGTTCTTATCG  R : AGTTAGGAGGACCGCAGGAATC  F : TTTCGATTCAGATGCTCC  R : CATCAGAGA GCC CCT GAA AG  F :GGCTCGGACTGTGATTGGTA  R : ACATGCAGTTCATCACTGTGG |
| *VvSWEET7*  *VvSWEET10*  *VvSWEET11*  *VvSWEET15*  *VvSWEET17a*  *VvSWEET17d*  *VvActin* | GSVIVT01019601001  GSVIVT01008595001  GSVIVT01010993001  GSVIVT01000938001  GSVIVT01035138001  GSVIVT01031170001  BN000705 | F :ACCGCAGTTGGCATCCTA  R : GCAAGGTAGGGAACTGGTGA  F : CCATTCACCATCCTTTGGTTT  R :CCACGTAGGGAACAGACTGAA  F :GGGACGTGCATAGAAGCTACA  R :GCAGACCCAACCGACTATCTT  F : GGCCAAGAAACAAACTCTCAAA  R :GCCACTGAGAATGAAGCACAG  F : GGTTTTGGTGTGGTTGTTGAA  R : AGCTAGAAACCCCACATCCAA  F : CTGGCGGCTTACTTGTCCT  R : AAAGCCAACATCCAATACGG  F : AGCTGGAAACTGCAAAGAGCAG  R : ACAACGGAATCTCTCAGCTCCA |
