# Supplementary material for: Coexpression of Sucrose Synthase and the SWEET Transporter, Which Are Associated With Sugar Hydrolysis and Transport, Respectively, Increases the Hexose Content in Vitis vinifera L. Grape Berries
Source: Front Plant Sci. 2020 Apr 30;11:321. doi: 10.3389/fpls.2020.00321 (PMC7221319; doi:10.3389/fpls.2020.00321)
Supplement: Supplementary file 2 [file Table_2.docx]

**Coexpression of sucrose synthase and the SWEET transporter, which are associated with sugar hydrolysis and transport, respectively, increases the hexose content in *Vitis vinifera* L. grape berries**

**Fronters in plant science**

Ruihua Ren^a^, Xiaofeng Yue^a^, Junnan Li^a^, Sha Xie^a^, Shuihuan Guo^a^, Zhenwen Zhang^a,b,*^

*Corresponding author: Zhenwen Zhang, College of Enology, Northwest A&F University, No. 22 Xinong Road, Yangling 712100, Shaanxi, China, Tel: 0086-13991879905; Email: [zhangzhw60@nwsuaf.edu.cn](mailto:zhangzhw60@nwsuaf.edu.cn).

**Supplemental Table 2** The sugar dynamic accumulation in in Riesling (RI), Petit Manseng (PM), Cabernet Sauvignon (CS) berries.

| Organs | Dates | DAA70 | | | DAA80 | | | DAA90 | | | DAA100 | | | DAA110 | | |
| --- | --- | --- | --- | --- | --- | --- | --- | --- | --- | --- | --- | --- | --- | --- | --- | --- |
|  | Genotypes | RI | PM | CS | RI | PM | CS | RI | PM | CS | RI | PM | CS | RI | PM | CS |
| Berries | Glucose（g/L） | 72.8 | 86.51 | 91.67 | 74.49 | 134.83 | 111.6 | 100.51 | 136.6 | 121.02 | 102.85 | 136.88 | 129.12 | 114.51 | 150.42 | 137.02 |
|  | Fructose（g/L） | 47.39 | 61.28 | 60.47 | 55.98 | 87.14 | 71.17 | 64.47 | 92.87 | 81.71 | 65.75 | 97.88 | 89.26 | 75.05 | 106.99 | 95.3 |
|  | Total:Hexose（g/L） | 120.19 | 147.80 | 152.14 | 130.47 | 221.97 | 182.77 | 164.98 | 229.47 | 202.72 | 168.59 | 234.76 | 218.37 | 189.56 | 257.41 | 232.31 |
