# Supplementary material for: Coexpression of Sucrose Synthase and the SWEET Transporter, Which Are Associated With Sugar Hydrolysis and Transport, Respectively, Increases the Hexose Content in Vitis vinifera L. Grape Berries
Source: Front Plant Sci. 2020 Apr 30;11:321. doi: 10.3389/fpls.2020.00321 (PMC7221319; doi:10.3389/fpls.2020.00321)
Supplement: Supplementary file 3 [file Table_3.docx]

**Coexpression of sucrose synthase and the SWEET transporter, which are associated with sugar hydrolysis and transport, respectively, increases the hexose content in *Vitis vinifera* L. grape berries**

**Fronters in plant science**

Ruihua Ren^a^, Xiaofeng Yue^a^, Junnan Li^a^, Sha Xie^a^, Shuihuan Guo^a^, Zhenwen Zhang^a,b,*^

*Corresponding author: Zhenwen Zhang, College of Enology, Northwest A&F University, No. 22 Xinong Road, Yangling 712100, Shaanxi, China, Tel: 0086-13991879905; Email: [zhangzhw60@nwsuaf.edu.cn](mailto:zhangzhw60@nwsuaf.edu.cn).

**Supplemental Table 3** The relative expression level of *SWEET*s in the Riesling (RI), Petit Manseng (PM), and Cabernet Sauvignon (CS) berries.

| Dates | DAA70 | | | DAA80 | | | DAA90 | | | DAA100 | | | DAA110 | | |
| --- | --- | --- | --- | --- | --- | --- | --- | --- | --- | --- | --- | --- | --- | --- | --- |
| Genes | RI | PM | CS | RI | PM | CS | RI | PM | CS | RI | PM | CS | RI | PM | CS |
| *SWEET4* | 0.082 | 3.4E-4 | 0.011 | 4E-5 | 2.1E-4 | 2.9E-4 | 3E-5 | 3E-5 | 3E-5 | 2E-5 | 6E-5 | 6E-5 | 8E-5 | 7.5E-4 | 9E-5 |
| *SWEET7* | 0.215 | 2E-4 | 0.003 | 2E-4 | 6.5E-4 | 0.001 | 9E-5 | 7E-5 | 6E-5 | 4E-5 | 1.3E-4 | 4E-5 | 5E-5 | 0.003 | 9E-5 |
| *SWEET10* | 0.066 | 0.238 | 0.326 | 0.220 | 0.073 | 0.265 | 0.181 | 0.012 | 0.271 | 0.106 | 0.005 | 0.182 | 0.069 | 0.001 | 0.043 |
| *SWEET11* | 0.124 | 1.9E-4 | 0.007 | 6.8E-5 | 1.4E-4 | 2.5E-4 | 2.7E-4 | 9.9E-5 | 0.001 | 0.001 | 0.002 | 0.001 | 0.002 | 5.702 | 0.001 |
| *SWEET15* | 0.175 | 1.312 | 0.471 | 0.119 | 4.323 | 1.600 | 0.961 | 2.856 | 1.499 | 0.330 | 1.479 | 0.873 | 0.373 | 2.910 | 1.197 |
| *SWEET17a* | 0.523 | 0.004 | 0.060 | 0.002 | 0.014 | 0.002 | 5.5E-4 | 5.6E-4 | 7.2E-4 | 0.002 | 0.001 | 5.7E-4 | 0.001 | 0.004 | 0.001 |
| *SWEET17d* | 0.028 | 4.3E-4 | 0.009 | 2.2E-4 | 7.3E-4 | 0.002 | 8.4E-5 | 7.3E-5 | 2.1E-4 | 3.8E-4 | 2.7E-4 | 7.2E-5 | 8.6E-4 | 0.001 | 3.1E-4 |
